# COVID-19 in South Africa: outbreak despite interventions

**DOI:** 10.1038/s41598-021-84487-0

**Published:** 2021-03-02

**Authors:** Malte Schröder, Andreas Bossert, Moritz Kersting, Sebastian Aeffner, Justin Coetzee, Marc Timme, Jan Schlüter

**Affiliations:** 1 Chair for Network Dynamics, Cluster of Excellence Physics of Life, Institute for Theoretical Physics and Center for Advancing Electronics Dresden (cfaed), Technical University of Dresden, Helmholtzstr. 18, 01069 Dresden, Germany; 2 Department of Social Sciences, Center of Methods in Social Sciences, Georg August University Göttingen, Goßlerstraße 19, 37073 Göttingen, Germany; 3 Next Generation Mobility Group (NGM), Department of Dynamics of Complex Fluids, Max-Planck-Institute for Dynamics and Self-Organization, Am Fassberg 17, 37077 Göttingen, Germany; 4 Institute for Diagnostic and Interventional Radiology, University Medical Center Göttingen, Robert-Koch-Straße 40, 37075 Göttingen, Germany; 5 GoMetro, 10 Church Street, Durbanville, Cape Town, 7550 South Africa; 6 Faculty of Physics, Institute for the Dynamics of Complex Systems, Georg August University of Göttingen, Friedrich-Hund-Platz 1, 37077 Göttingen, Germany; 7 Lakeside Labs, Lakeside Park B04, 9020 Klagenfurt, Austria

**Keywords:** Health policy, Complex networks, Nonlinear phenomena

## Abstract

The future dynamics of the Corona Virus Disease 2019 (COVID-19) outbreak in African countries is largely unclear. Simultaneously, required strengths of intervention measures are strongly debated because containing COVID-19 in favor of the weak health care system largely conflicts with socio-economic hardships. Here we analyze the impact of interventions on outbreak dynamics for South Africa, exhibiting the largest case numbers across sub-saharan Africa, before and after their national lockdown. Past data indicate strongly reduced but still supracritical growth after lockdown. Moreover, large-scale agent-based simulations given different future scenarios for the Nelson Mandela Bay Municipality with 1.14 million inhabitants, based on detailed activity and mobility survey data of about 10% of the population, similarly suggest that current containment may be insufficient to not overload local intensive care capacity. Yet, enduring, slightly stronger or more specific interventions, combined with sufficient compliance, may constitute a viable option for interventions for South Africa.

## Introduction

The severe acute respiratory syndrom coronavirus 2 (SARS-CoV-2) has reached more than 200 countries and territories across all continents^1, 2^. By death toll, the resulting Corona Virus Disease 2019 (COVID-19) outbreak will likely soon become the largest pandemic of the 21st century so far^3^. There is currently no specific medical intervention known against SARS-CoV-2 and preventive vaccination options are not yet available. The resulting vast number, broad geographical distribution, and intensity of globally enacted socio-economic interventions is unprecedented in modern human history.

Mainland China was the first region hit by the outbreak in January 2020 and had taken rapid and severe interventions including an almost complete lockdown for 11 weeks. It thereby succeeded to suppress the outbreak dynamics to subexponential growth patterns^4^ and in April 2020 is reporting a total of 83,500 cases and at most 130 new cases daily for now more than 5 weeks^2^. As of April 30th, several countries in Europe are reporting more than 100,000 cases each and the United States alone reports above 1 Million cases.

At the same time, Africa as a continent with a population of 1.3 billion people (as of 2018 ^5^) has reported only about 24,000 cases^2, 6, 7^. Of those, the largest number of COVID-19 patients is reported in South Africa with about 5300 cases and 100 (about 1.9%) deaths as of April 30, 2020^2, 8^. Across all these countries, the total number of cases is increasing. Due to heterogeneous conditions and often broadly undersampled testing and reporting, the future outbreak dynamics in Africa remains largely unclear.

Across the African continent, national economic constraints, individual poverty, low health literacy rates, weaker health care systems and cultural practices lead to reduced option spaces for interventions on personal and governmental levels and may all contribute to more severe consequences of the COVID-19 outbreak and negatively influence containment as well as recording, testing and medical treatment^9^. Similar conditions will hold for most countries of the Global South, calling for particular attention on African countries^10^.

In general, health care systems in African countries feature only a small number of available intensive care units (ICUs) compared to most countries of the Global North^11, 12^. At the same time, African countries are under particular pressure due to economic constraints. Besides strong repercussions on national economic productivity expected for any large-scale lockdown, a large fraction of the population is unable to fully comply with severe lockdown measures due to their personal financial or social situation. An African task force for coronavirus preparedness and response (AFTCOR) has been established to manage these combined and conflicting constraints both for the current COVID-19 outbreak and for future preparedness^13^. Their work focuses on enabling medical diagnosis and screening options, clinical treatment of COVID-19 patients, infection prevention and control in health care facilities, supply chain management, and the communication of risks to experts and the public. Qualitative and quantitative time series analysis on reported cases in Africa and estimates of the future outbreak dynamics by evaluating implications of containment options essentially underlie but are not in the focus of their work.

South Africa offers a comparatively high capacity of intensive care units (ICUs) to respond to outbreaks, with estimates ranging from maximally 7195 ICU beds theoretically in existence to 2926 practically available nationwide across both public and private sectors^14^. The order of magnitude of these numbers is consistent with earlier reports^15^. However, the factually available ICU beds have likely declined during the past decade necessitating rationing and triage (prioritisation) decisions that have been frequently necessary in South Africa even in times before COVID-19, particularly in the publicly funded health sector^14, 16^. Moreover ICU capacity in the private sector is not readily and generally accessible.

## Results

### Influence of lockdown on past case numbers

On March 5, 2020, the first COVID-19 patient has been confirmed in South Africa and after starting with specific smaller measures from March 15 onwards, the South African government enacted a national lockdown effective March 27, 2020. This lockdown includes measures such as the complete closure of *childcare*, institutions of primary and higher *education* as well as all public *leisure* activities, severe *physical distancing* rules, an estimated 70% reduction of *shopping*, 85% of on-site *work* force and a 90% reduction in *other* activities. An initial formal reduction of shared publicly available mobility services by about 75% was, after protests, revised to about 30% reduction^17^ (estimates by GoMetro, South Africa). These shared mobility services provide a large fraction of transportation and constitute one of the special conditions in South Africa and many other African countries^18^. For instance in South Africa, instead of formal public transit, transportation is dominated by private, semi-regulated minibus taxis with typically 15 seats^18^. Due to their mass usage, usually high occupancy and the close contact between passengers in the vehicles, these mobility services may contribute substantially to the spread of COVID-19.

**Figure 1:**
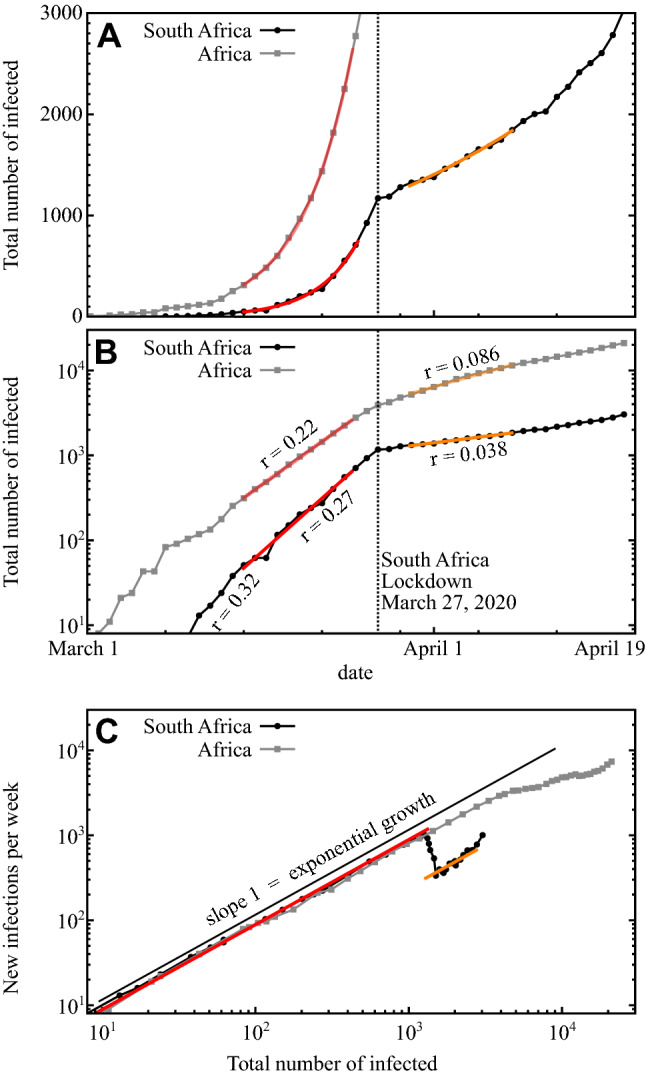
COVID-19 in Africa and South Africa. (**A**,**B**) The number of confirmed COVID-19 patients in Africa (gray squares) and specifically South Africa (black disks) from March 1, 2020 until April 16, 2020, on (**A**) linear and (**B**) logarithmic scales. Best exponential fits (colored lines) yield growth rates *r* where the total number of (reported) infected patients $N \propto \exp (r \, t)$ where *t* measures time in days. (**C**) State space representing the number of newly reported patients as a function of the total of reported people infected (including the recovered), eliminating absolute time. Straight solid line of slope 1 indicates pure exponential growth. The impact of the lockdown executed on March 27 is clearly visible (vertical lines in (**A**,**B**)).

Fitting the number of total reported cases in South Africa before and after the national lockdown (Fig. 1) indicates that the lockdown drastically reduces the relative increase in case numbers, as quantified by the growth exponent, decreasing from $r=0.32$ per day in the beginning of the outbreak to about $r=0.27$ per day just before the lockdown and down to $r=0.038$ per day after the lockdown, reflecting an increase of the doubling time from about 2.5 to about 18 days (Fig. 1A,B). The immediate switch to slower growth at the date of the official lockdown may be originating from several factors the detailed influence of which remain unknown.

As the number of cases in South Africa makes up a substantial share of all reported cases throughout Africa, the effect also becomes visible in the data for the entire continent (Fig. 1A,B). For Africa as a whole, growth exponents dropped from about $r=0.22$ to $r=0.086$. The data for Africa suggest a further decrease of the exponent, ongoing after the South African lockdown.

While the growth exponents have been substantially reduced, between a factor of 7.1 (South Africa) and a factor of 2.6 (all of Africa), the growth remains exponential at least 3 weeks into the lockdown. This is in stark contrast to the outbreak dynamics in Mainland China, where the strict containment measures of the Hubei region has led to subexponential growth^19^ followed by a massive decrease of new case numbers within weeks after lockdown^2^. The initially unbroken exponential growth trend in South Africa is also indicated by the number of newly infected people per week steeply increasing when displayed as a function of the total number of infected (Fig. 1C), instead of curving down.

### Modeling future scenarios

The current national lockdown has been extended from an original three weeks (until April 17, 2020) with relaxations now suggested for the beginning of May, 2020. We thus ran scenario simulations to estimate future case numbers and probe responses to different intervention strengths and durations. We employed a computational data-driven, agent based transport model for the Nelson Mandela Bay Municipality (NMBM, Eastern Cape, South Africa, 1.14 million inhabitants)^20^ with lockdown fractions of work, leisure, and shopping activities and complete lockdown of childcare and educational institutions, in line with measures currently implemented in South Africa. To reflect potential non-compliance with enacted lockdown measures, the simulations took only a 85% reduction of *other* activities; for minibus taxi services we took a 50% effective reduction of passengers, to reflect the tradeoff between non-compliance and the reduction in demand due to less people required or wishing to travel caused by the other lockdown measures and the outbreak. The parameter assumptions are based on mobility data provided by the South African local mobility business GoMetro (see “Methods” section for further details of model setup).

**Figure 2:**
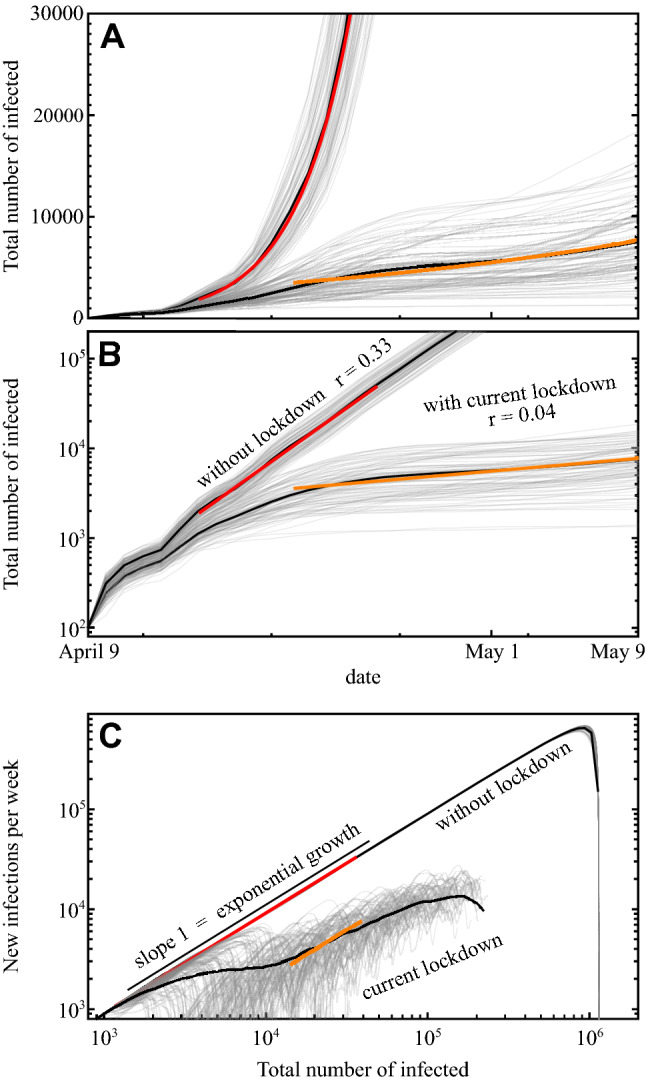
Estimated COVID-19 cases for the Nelson Mandela Bay Municipality, South Africa. (**A**,**B**) Simulation of the outbreak without interventions (red fit) and with current interventions (orange fit) on (**A**) linear and (**B**) logarithmic scales. Thin grey lines represent individual simulations, the solid black lines represent their averages. Growth rates are consistent with the observations for South Africa in the beginning of the outbreak (without lockdown) and after the lockdown (compare Fig. 1). (**C**) State space representing the number of newly reported patients as a function of the total of reported people infected (including the recovered), eliminating absolute time. While the lockdown measures slow the spread of the outbreak, the growth remains exponential for some time (compare also Fig. 1C).

**Figure 3:**
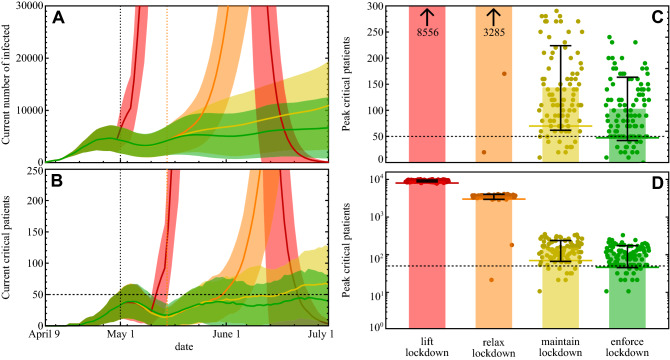
Influence of intervention policies. (**A**) Number of active infections over time. Solid lines indicate averages across realizations, shaded areas indicate standard deviation. Color encodes the four scenarios: lockdown lifted May 1st (red), lockdown relaxed by 25% on May 15 (orange), maintain current lockdown until June 30 (yellow), and enforcing lockdown or increasing compliance from May 1, 2020 (green). (**B**) Number of critical patients and estimated capacity available in NMBM (horizontal dotted line). Data encoding as in (**A**). The dashed vertical line illustrates the available ICU capacity. (**C**,**D**) Maximum number of patients requiring intensive care during the outbreak until end of June 2020, across scenarios (color code as before). Bars indicate averages across realizations and standard deviation, small disks individual realizations. The solid lines indicate the peak of the average trend, which is below the peaks of the individual realizations as these peaks may occur at different times. Note that these numbers may increase after June 2020, for example when maintaining the lockdown (compare upwards trend in (**B**)). All data based on 100 realizations of agent-based simulations for each of the four scenarios for NMBM, South Africa.

Calibrating our simulations to the growth rate before lockdown ($r=0.33 \pm 0.02$ average and standard deviation over 100 realizations), our results with the estimated restirctions are consistent with the growth exponents of the total number of infected individuals after national lockdown ($r=0.04 \pm 0.02$ average and standard deviation over 100 realizations), see Fig. 2A,B. The exponents cannot be specified more exactly due to the unpredictable stochastic factors in the transmission process creating substantial variations in particular at low case numbers, sampled over in simulations with one hundred random realizations each. Importantly, there are simulated case dynamics that display an early (within April, 2020) saturation of the total number of cases at 10,000 or below. However, the ensemble of simulations of the lockdown scenario suggests an ongoing outbreak either entirely without saturation or with early but non-persistent saturation and renewed increase, likely in May. Figure 2C displays the same data of the dynamics in a state space characterizing the epidemics without referring to absolute time (as in Fig. 1C), thereby enabling to compare system-wide potential pathways. The results illustrate that current lockdown measures substantially slow the spread of the outbreak in all realizations, but only in 4 out of 100 realizations the outbreak ends before 10,000 people become infected in the Nelson Mandela Bay Municipality alone.

To evaluate the expected outbreak dynamics and the maximal number of critical patients requiring intensive care, we studied four different scenarios by agent-based simulations, again 100 realizations per scenario (Fig. 3). Entirely lifting the currently enacted lockdown on May 1 would cause an immediate rise of infected patient numbers and a delayed rapid rise of critical patient numbers drastically beyond the ICU capacity available in NMBM (estimated to be 50 based on downscaling (proportional, by population size) the 267 ICU beds expected to be available in the entire Eastern Cape Province^21^). Whereas the exact numbers will depend on details of the simulation, further simulations (not shown) indicate a manifold overload of ICU capacity also after varying mobility parameters. Lifting lockdown by 25% two weeks later, i.e. on May 15, still would cause massive rise in case numbers and ICU overload in early June. Maintaining current lockdown conditions strongly slows the outbreak, yet our simulations suggest that such interventions together with current compliance are marginally insufficient to contain the epidemic long term and keep the number of critical patients below ICU capacity (Fig. 3B,C), as suggested already by our data analysis of past case numbers (Fig. 1). Finally a fourth scenario of slightly strengthening current interventions, either by slightly stricter, possibly even more specific lockdown regulations, by increasing compliance, or a combination of both (90% reduction of shopping and other, 95% reduction of work activities and complete restriction of all other activities including public mobility in the simulations), may keep the number of critical COVID-19 patients at or below the ICU capacity and may largely contain the epidemic by end of June 2020.

## Discussion

The analysis of reported past case data is robust and suggests that the outbreak currently still grows too quickly to contain the number of critical COVID-19 patients significantly below available ICU capacities nation-wide. Observations like the immediate downtrending when the lockdown comes into effect in South Africa may be potentially explained by, e.g., the number of patients tested per day having substantially increased initially^8, 22^ or tests having potentially been delayed at the very onset. In any given region, the first person infected is likely detected only after exhibiting symptoms while later cases may be identified by preemptive contact tracing and thereby identified as they appear, ideally before showing symptoms. Other contributing factors may include stochastic small number fluctuations occurring at the onset of any epidemic outbreak, and already existing awareness of the COVID-19 outbreak and countermeasures taken before the official national lockdown.

The continuous downtrending of the growth rate across all of Africa may be associated with measures taken up at different points in time in the most strongly affected countries of Northern Africa, and the vastly heterogeneous case numbers, test coverage and reporting of cases across African countries, all entangling with the reduced number, but still large share of South African COVID-19 patients. The main potential causes of errors in the analysis of past data may be biased or undersampled testing and reporting of case numbers.

Predicting future case numbers and the number of critical patients under different scenario conditions is much more difficult. The most difficult challenge is the bridging of scales between known or estimated country-wide overall conditions and specific urban level scenarios (at 1.14 million people) that are again subsampled at about 10% of the population, *not* primarily due to simulational constraints but due to the availability of socio-economic and travel data for about 100,000 people only^20^. Combined with the COVID-19 outbreak being at an early stage, the number of infected patients is of an order of magnitude between $10^1$ and $10^3$ in NMBM, thereby causing strong stochastic number fluctuations that make individual predictions unreliable. We attempted to compensate for such fluctuations partially by running ensemble simulations for 100 random realizations, with a random subsample of initial patients infected (and thus varying their location, household size, employment status etc.). As the results are based on limited ensemble simulations, they likely underestimate the probability of extreme outcomes such as strong increase or random decay of the outbreak.

The results reported above suggest that current lockdown levels may be just marginally insufficient to prevent a massive COVID-19 outbreak in South Africa. As the increase in case numbers is still exponential and not subexponential as reported for Mainland China^19^, South Africa may be still in the unfortunate situation to become for the African continent what Italy has been for Europe^23^, with potentially devastating consequences.

A rapid large-scale infection within weeks to a few months, the likely outcome if the national lockdown was lifted or relaxed early May^8^, implies a manifold overload of ICU capacity. Interventions slightly stronger than those implemented today, or even a higher degree of compliance to the enacted lockdown alone may constitute a viable chance for effective countermeasures for regions in South Africa and potentially for large parts of the African continent.

The current model setting does not explicitly include demographical resolution of the population in the modelling of the disease progression because data sets of behavioral and activity patterns are not available in a demographically resolved way. The population of agents and their activities still represents an accurate sample across the whole demographic range. While we expect the explicit modelling of different demographics to quantitatively modify our results, the very nature of the transition observed (from decreasing to rapidly increasing case numbers depending on the severity of countermeasures) is robust against any such detailed changes. Future studies for this or other regions with available detailed demography data may shine further light on the detailed influence of demography and its correlation to activity patterns, potentially with spatial resolution at the level of city quarters.

However, a number of boundary conditions beyond those known for past major hubs of the COVID-19 pandemic in countries like Mainland China, the United States or Italy^23^ need to be taken into account simultaneously. Most African countries find themselves under much stronger socio-economic and health care system constraints than countries of the Global North.

For instance, a large fraction of the work force both is at lower-income levels and simultaneously has no fall-back option to remote work. As many of such work activities are not tagged “essential” in the sense of the lockdown, people often have zero income or immediately fall into extreme poverty. Moreover, even where remote work is possible, it comes with additional challenges^24^. Still, South Africa is potentially in a better position than many other African countries, so the conclusions (for South Africa specifically) might be conservative in this sense.

The South African health situation includes a high risk of COVID-19 coinfections for patients with, e.g., HIV/AIDS or forms of tuberculosis (TBC). It implies additional challenges, which are concerned to have a detrimental effect on the criticality of COVID-19 infections or medcine and health care supply^25–27^. According to the WHO 2019, South Africa ranks 4th globally in the number of TBC infections *per capita* and 3rd for those coinfected with TBC and HIV. Moreover, the South African population infected with TBC alone is about 320,000 (0.5%, about 20 times higher rate than in Europe) and a total of 7.7 million people (13%) are infected with HIV as of 2018^28, 29^.

Regulatory decision against COVID-19 cannot only take care of short-term economic constraints^17^. A large-scale outbreak and massive ICU overload may have drastic consequences for the country as a whole, including societal and economic but also psychological, and ethical issues (compare^30^). Thinking of economic constraints should also imply of long-term implications, for both economy and society. This perspective underlines again the coaction advocated by the United Nation’s Sustainable Development Goals (SDGs), in particular Good Health and Wellbeing (SDG 3), Sustainable Cities and Transportation (SDG 11), and Reduced Inequality both within and among countries (SGD 10) in the context of COVID-19.

An integrated perspective on such goals may help paving the way to a fair and sustained solution of the COVID-19 crisis and future pandemics across African countries as well as for individuals, groups and regions in a position much more fragile than common for countries of the Global North, as also underlined by the proposed CoHERE programme^31^.

Finally, our results indicate that large-scale agent-based simulations integrating microscopic mobility and activity data on the individual person level and for areas with $10^6$ or more inhabitants, in combination with fundamental nonlinear and stochastic dynamics analysis may serve as a valuable tool of qualitatively predicting longer-term outcomes of epidemic spreading dynamics under a variety of scenarios.

## Methods

### Data

#### Sources

All data is based on the aggreagated COVID-19 case numbers collected by Johns Hopkins University^2^, downloadable from https://github.com/CSSEGISandData/COVID-19/tree/master/csse_covid_19_data/csse_covid_19_time_series (accessed at 20-04-20). The data provides the total number of confirmed (reported) cases per day and per country (for Africa, higher resolution is available for some other countries). We aggregate the available country-level data for all African countries to compute the per-day case numbers for Africa shown in Fig. 1.

#### Analysis of COVID-19 case number dynamics

We compute the growth rates *r* from the data based on linear regression of the logarithm of the total number of confirmed cases in a ten day interval based around the official beginning of the lockdown in South Africa. The fits shown in Fig. 1 are only shown in the interval where they were performed, before lockdown in the interval 15-03-20 to 25-03-20 (up to two days before the lockdown, $r=0.27$), after the lockdown in the interval 30-03-20 to 09-04-20 (beginning three days after the lockdown, $r=0.038$). For comparability, we use the same intervals to compute the growth rate of cases in Africa as a whole.

We remark that the instantaneous growth rates change over time, especially before the lockdown. The initial growth rate in South Africa reported in the main text and noted in Fig. 1 is based on a fit in the interval 10-03-20 to 20-03-20 with higher growth rate ($r=0.32$). Uncertainties of the reported results of the regression are much smaller ($R^2 > 0.99$, relative error of the growth rate less than $3\%$) than the variation of the growth rate over time.

### Simulation

#### Basics of data-driven agent-based model

The simulation results reported in Figs. 2 and 3 are based on a detailed agent-based simulation of 10% of the population (114,346 agents, such that each agent represents 10 people) of NMBM conducted with MATSim (version 12.0-SNAPSHOT)^32^. The (synthetic) population of agents that serves as input for the MATSim simulation is that suggested by Joubert et al.^33^, aggregating survey data on social and economic conditions as well as detailed travel diaries. In the simulation, each agent chooses a transport mode and route based on their activity schedule (type and place of planned activities such as work, school or shopping). This process is repeated until the agents route choice settles into a (statistically) stationary state.

We adjusted the standard MATSim framework to account for the high prevalance of semi-formal publicly accessible transport options via minibus taxis^18^. We model this transport mode via a model for Demand Responsive Transport (DRT) for MATSim^34^, allowing the agents to call a dynamically routed bus to specific stop locations. As this model does not perfectly reproduce the minibus taxi system as it typically operates in South Africa, we adjust the fleet size (2300 buses) of these minibus taxis and their capacity (15 seats) to reproduce the actually observed utilization (2374 minibus taxis with an official average capacity of 15 passengers in 2014 in NMBM^35^).

The resulting contact network (describing potential interactions of agents with one another during their activities and commutes) from the MatSim simulation is then used as input for the EpiSim simulation^36^ (https://github.com/matsim-org/matsim-episim, master, accessed April 11, 2020), modelling the epidemic spreading on this contact network. The probability $P_{n,t}$ for an agent *n* to be infected during an activity that ends at time *t* is based on the other (infectious) agents *m* it is in contact with, the duration of exposure $\tau _{nm,t}$, the (relative and constant) shedding rate $q_{m,t}$ with which a infectious agent distributes the virus, and the (relative) intensity $i_{m,t}$ of contact during the activity such that

1 $$\begin{aligned} P_{n,t}&= 1- \exp [-\theta \sum _{m} q_{m,t} i_{nm,t} \tau _{nm,t}]\,. \end{aligned}$$

The free parameter $\theta$ is used to calibrate the model to fit the observed growth rate. Here, we take $\theta = 1.5\times 10^{-6}$ to match the uninhibited growth rate observed $r_{\text {max}} = 0.32$ in the case data for South Africa.

The activities considered in the EpiSim model are *Home*, *Work*, *Primary Education*, *Higher Education*, *Shopping*, *Leisure*, *Dropby*, *Other* and *Minibus taxi commute*. Agents do not risk infection by commuting by private car (if available) or walking. Here, *work*, *shopping* have the same contact intensity as *leisure*, $i_{\text {leisure}} = 5$. The activities *dropby* and *other* have contact intensities $i_{\text {dropby}} = 7$ and $i_{\text {other}} = 3$, respectively. To account for the specific condition in South Africa compared to the original location (Berlin, Germany) that the model was developed for, we adjusted several parameters affecting the intensity of contact during specific activities. In particular, we increased the contact intensity for the activities *home* to $i_{\text {home}} = 6$ (compared to 3 in the original EpiSim setting) due to general living conditions and the contact intensity during commutes with minibus taxis to $i_{MBT} = 20$ (compared to 10 for public transport in the original EpiSim setting). Other parameters are left as in the original EpiSim setting^36^, such that *susceptible* agents first become *infected but not contagious*, then *contagious* after four days, where $20\%$ of the affected agents self-quarantine on day 6 for the duration of the illness and cannot infect any other agents. These infected agents either recover after a total of 16 days or become *seriously sick* after after a total of 10 days (4.5% probability) and potentially *critical* on the next day (20% of all seriously sick agents). These cases terminate after a total of 23 days.

All simulations start with 10 infectious agents (day 4 of the course of the disease) on 09-04-20. These agents are selected uniformly at random from the total population.

#### Scenarios

The scenarios explored in Fig. 3 adjust the share of agents that perform a certain activity by different factors (see also main text). In the lift lockdown scenario, all activities are unrestricted after May 1, described by an activity factor of $\alpha = 1$ (100%) for all activities. Accordingly, reducing the $\alpha$ of an activity to 0.5 would exclude 50% of the agents who would normally carry out that activity.

The current lockdown conditions are described by a *work* activity factor $\alpha _{\text {work}} = 0.15$ (85% reduction), $\alpha _{\text {shopping}} = 0.3$, $\alpha _{\text {other}} = 0.15$ and a complete shutdown of childcare, education and leisure and dropby activities ($\alpha = 0$). Additionally, the minibus taxi activity was reduced by $50\%$, $\alpha _{MBT} = 0.5$.

To model an enforced lockdown (or higher compliance with prescribed rules), we restrict shopping and other activities to $\alpha _{\text {shopping}} = \alpha _{\text {other}} = 0.1$, work to $\alpha _{\text {work}} = 0.05$ and completely shut down public mobility, $\alpha _{MBT} = 0$. To model a relaxed lockdown, we assume that all activity restrictions are reduced by $50\%$ such that $\alpha _{\text {relax}} = 1 - (1-\alpha _{lockdown})/2$, based on the current restrictions.

#### Scenario-based estimates

All results reported in Figs. 2 and 3 show the average from 100 realizations (random initial conditions and stochastic infection process). Growth rates of the simulation results are obtained from linear regression of the average evolution in the intervals 15-04-20 to 25-04-20 (without lockdown) and 20-04-20 to 10-05-20 (with lockdown). The reported uncertainty (see main text) is the standard deviation of the computed growth rate in the same interval for the individual realizations.

To compute the average number of new infections per week as a function of the total number of infected, we averaged over identical values of the total number of infected (instead of over time), where we combine data into logarithmic bins $[n, 1.1\,n]$, starting at $n_{\text {min}} = 10$ infected.

The peak critical patients reported in Fig. 3 indicate the global maximum of the number of (concurrent) critical patients in the simulation until July 1. We note that this does not necessarily represent the global maximum over all time as case number may still increase after this date, in particular in the relax and maintain lockdown scenarios.

## Data Availability

The population data analysed during the current study are available under https://doi.org/10.17632/dh4gcm7ckb.1. The epidemic simulation framework used during the current study is available under https://github.com/matsim-org/matsim-episim.
